# An empirical staging model for schizophrenia using machine learning

**DOI:** 10.1192/j.eurpsy.2023.1304

**Published:** 2023-07-19

**Authors:** M.-C. Clara, F. Sánchez-Lasheras, A. García-Fernández, L. González-Blanco, P. A. Sáiz, J. Bobes, M. P. García-Portilla

**Affiliations:** 1 Department of Psychiatry, University of Oviedo; 2 Instituto de Investigación Sanitaria del Principado de Asturias (ISPA); 3 Instituto Universitario de Neurociencias del Principado de Asturias (INEUROPA); 4 Department of Mathematics, University of Oviedo; 5 Instituto Universitario de Ciencias y Tecnologías Espaciales de Asturias (ICTEA), Oviedo, Asturias; 6 Centro de Investigación Biomédica en Red de Salud Mental (CIBERSAM); 7 Servicio de Salud del Principado de Asturias (SESPA), Oviedo, Spain

## Abstract

**Introduction:**

One of the great challenges still to be achieved in schizophrenia is the development of a staging model that reflects the progression of the disorder. The previous models suggested have been developed from a theoretical point of view and do not include objective variables such as biomarkers, physical comorbidities, or self-reported subjective variables (Martinez-Cao *et al.* Transl Psychiatry 2022; 12(1) 1-11).

**Objectives:**

Develop a multidimensional staging model for schizophrenia based on empirical data.

**Methods:**

Naturalistic, cross-sectional study. Sample: 212 stable patients with Schizophrenia (F20). Assessments: *ad hoc* questionnaire (demographic and clinical information); psychopathology: PANSS, CDS, OSQ, CGI-S; functioning: PSP; cognition: MATRICS; laboratory tests: C-Reactive Protein (CRP), IL-1RA, IL-6, Platelets/Lymphocytes (PLR), Neutrophils/Lymphocytes (NLR), and Monocytes/Lymphocytes (MLR) ratios. Statistical analysis: Variables selection was performed with an *ad hoc* algorithm developed for this research. The referred algorithm makes use of genetic algorithms (GA) to select those variables that show the best performance for the patients classification according to their global CGI-S. The objective function of the GA maximizes the individuals correct classification of a support vector machines (SVM) model that employs as input variables those given by the GA (Díez-Díaz *et al.* Mathematics 2021; 9(6) 654). Models performance was assessed with the help of 3-fold cross-validation and these process was repeated 10,000 times for each one of the models assessed.

**Results:**

Mean age(SD): 39.5(13.54); men: 63.5%; secondary education: 59.50%. Most patients in our sample had never been married (74.10%), and more than a third received disability benefits due to schizophrenia (37.70%). The mean length of the disease was 11.98(12.02) years. The best SVM model included the following variables: 1)Clinical: number of hospitalizations, positive, negative, depressive symptoms and general psychopathology; 2)Cognition: speed of processing, visual learning and social cognition; 3)Functioning: PSP total score; 4)Biomarkers: PLR, NLR and MLR. This model was executed again 100,000 times applying again 3-fold cross-validation. In 95% of the algorithm executions more than a 53.52% of the patients were classfied in the right CGI-S category. On average the right classification was of 61.93%. About specificity and sensitivity the average values obtained were of 0.85 and 0.64 respectively.

**Conclusions:**

Our staging model is a robust method that appropriately distributes patients according to the severity of the disorder. Highlights the importance of clinical, functional and cognitive factors to classify patients. Finally, the inflammatory parameters PLR, NLR and MLR have also emerged as potential biomarkers for staging schizophrenia.

**Disclosure of Interest:**

None Declared

